# Niclosamide does not modulate airway epithelial function through blocking of the calcium activated chloride channel, TMEM16A

**DOI:** 10.3389/fphar.2023.1142342

**Published:** 2023-03-06

**Authors:** Henry Danahay, Sarah Lilley, Kathryn Adley, Holly Charlton, Roy Fox, Martin Gosling

**Affiliations:** ^1^ Enterprise Therapeutics Ltd., Brighton, United Kingdom; ^2^ Sussex Drug Discovery Centre, School of Life Sciences, University of Sussex, Brighton, United Kingdom

**Keywords:** calcium activated chloride channel, channel blocker, ion channel, ion transport, goblet cell, mucus, mucin

## Abstract

Niclosamide and benzbromarone have been described as inhibitors of the calcium activated chloride channel, TMEM16A, and on this basis have been considered and tested as clinical candidates for the treatment of airway diseases. However, both compounds have previously demonstrated activity on a range of additional biological targets and it is unclear from the literature to what extent any activity on TMEM16A may contribute to efficacy in these models of airway disease. The aim of the present study was therefore to examine the pharmacology and selectivity of these clinical candidates together with a structurally unrelated TMEM16A blocker, Ani9, in a range of functional assays to better appreciate the putative role of TMEM16A in the regulation of both epithelial ion transport and the development of an airway epithelial mucus secretory phenoptype. Benzbromarone and Ani9 both attenuated recombinant TMEM16A activity in patch clamp studies, whereas in contrast, niclosamide induced a paradoxical potentiation of the TMEM16A-mediated current. Niclosamide and benzbromarone were also demonstrated to attenuate receptor-dependent increases in intracellular Ca^2+^ levels ([Ca^2+^]_i_) which likely contributed to their concomitant attenuation of the Ca^2+^-stimulated short-circuit current responses of FRT-TMEM16A and primary human bronchial epithelial (HBE) cells. In contrast, Ani9 attenuated the Ca^2+^-stimulated short-circuit current responses of both cell systems without influencing [Ca^2+^]_i_ which supports a true channel blocking mechanism for this compound. Additional studies using HBE cells revealed effects of both niclosamide and benzbromarone on global ion transport processes (absorptive and secretory) as well as signs of toxicity (elevated LDH levels, loss of transepithelial resistance) that were not shared by Ani9. Ani9 also failed to influence the IL-13 induced differentiation of HBE towards a goblet cell rich, mucus hypersecreting epithelium, whereas niclosamide and benzbromarone attenuated numbers of both goblet and multiciliated cells, that would be consistent with cellular toxicity. Together these data challenge the description of niclosamide as a TMEM16A blocker and illustrate a range of off-target effects of both niclosamide and benzbromarone which may contribute to the reported activity in models of airway function.

## Introduction

The TMEM16A protein (anoctamin-1) has been established as a calcium activated chloride channel (CaCC) in both recombinant and native cell systems (Caputo et al., 2008; Schroeder et al., 2008; Yang et al., 2008). Physiological roles for TMEM16A protein function have been described in numerous processes including the regulation of ion transport, smooth muscle contraction, neuronal conduction and cell proliferation (reviewed in Pedemonte & Galietta, 2014). To this end, therapeutic opportunities may exist in the regulation of TMEM16A function for the treatment of a host of human diseases including cystic fibrosis, asthma, pulmonary hypertension, secretory diarrhoea and cancer.

Low molecular weight compounds have been identified which are reported as selective blockers of TMEM16A function and include: T16Ainh-A01, CaCCinh-A01, MONNA, niclosamide, benzbromarone and Ani9 (De La Fuente et al., 2008; Namkung et al., 2011; Huang et al., 2012; Seo et al., 2016; Miner et al., 2019). These compounds have been widely utilised to support the identification of the biological activities associated with TMEM16A. In the case of both benzbromarone and niclosamide, repurposing of these agents as inhibitors of TMEM16A for the treatment of pulmonary hypertension and respiratory diseases have been proposed and clinical studies performed (Cabrita et al., 2019; Miner et al., 2019; Papp et al., 2019; Singh et al., 2022).

The selectivity of some of these compounds for TMEM16A has however been recently questioned. For example, T16Ainh-A01, CaCCinh-A01, and MONNA were demonstrated to relax rodent arteries with a mechanism of action that was independent of CaCC activity (Boedtkjer et al., 2015). Additional pharmacological activities for these three compounds include inhibition of TMEM16B (ANO2) and volume-regulated anion channels (VRAC) (Seo et al., 2016) as well as effects on the regulation of intracellular Ca^2+^ ([Ca^2+^]_i_) homeostasis (Cabrita et al., 2017; Genovese et al., 2022). Understanding the selectivity of niclosamide and benzbromarone are important to enable the interpretation of the pre-clinical data supporting the repurposing of these agents.

The aim of the present study was to test the hypothesis that benzbromarone, niclosamide and Ani9 would all selectively block TMEM16A ion channel function and that this activity would be independent of effects on intracellular Ca^2+^ levels. Furthermore, the effects of these agents on models of airway epithelial function would be tested to support the utility of benzbromarone and niclosamide as potential candidates for therapeutic repurposing.

## Methods

Unless otherwise stated, all chemicals were purchased from Sigma (Poole, Dorset, United Kingdom) and cell culture/immunofluorescence reagents from Thermo Fisher Scientific (Inchinnan, Renfrew, United Kingdom).

### Cell culture

FRT-hTMEM16Aabc cells were provided by Dr Luis Galietta (Genoa, IT) and were cultured as previously described for either QPatch or ion transport studies (Caputo et al., 2008). In brief, cells were cultured in Coon’s modified Ham’s F-12 medium. Media was supplemented with 10% fetal calf serum, 2 mM L-glutamine, 100 U/mL penicillin, 100 μg/mL streptomycin, G418 (750 μg/mL) and sodium bicarbonate (0.26%). Cells were seeded onto polycarbonate Snapwell inserts (0.5 × 10^6^ cells/insert) and used between 4 and 10 days after seeding. Cells were fed every 48 h and the day before an ion transport experiment.

Human bronchial epithelial (HBE) cells were provided by Dr Scott Randell from both the University of North Carolina Chapel Hill collection and the Cystic Fibrosis Foundation Therapeutics repository. Normal and CF (CF-HBE) cells were cultured at air-liquid interface as previously described (Danahay et al., 2020a). In brief, following an expansion step on plastic, HBE were seeded onto either Snapwell or Transwell inserts in submerged culture followed by 2 weeks at air-liquid interface in DMEM:F12 media supplemented with Ultroser G (2% v/v; Pall, Cergy, France). At all stages of culture, cells were maintained at 37°C in 5% CO_2_ in an air incubator.

### Whole-cell patch clamp assay

Whole-cell voltage-clamp recordings from FRT-hTMEM16Aabc, HEK-hTMEM16Aacd, and HEK293 cells were made using the QPatch planar patch clamp system as described previously (Bill et al., 2015; Danahay et al, 2020a). TMEM16A currents were assessed using chloride selective solutions with calculated free [Ca^2+^]_i_ tightly buffered at either 415 or 0 nM. Current rectification ratios were calculated by dividing the magnitude of the outward current at +90 mV by the magnitude of the inward current at −90 mV.

### Short-circuit current (ISC) measurements

FRT-hTMEM16Aabc cells were mounted in Ussing chambers in asymmetrical Ringers solutions. The basolateral Ringers contained (in mM): 120 NaCl, 25 NaHCO_3_, 3.3 KH_2_PO_4_, 0.8 K_2_HPO_4_, 1.2 CaCl_2_, 1.2 MgCl_2_, and 10 glucose. In the apical Ringers solution, NaCl was reduced to 80 mM and 40 mM sodium gluconate was included to establish a Cl^−^ gradient. The solution osmolarity was between 280 and 300 mosmol kg H_2_O^−1^ and was continuously gassed (5% CO_2_ in O_2_; pH 7.4) and maintained at 37°C. Cells were voltage clamped to 0 mV (model EVC4000; WPI) and the short-circuit current (ISC) was measured. Data were recorded using a PowerLab workstation and LabChart software (ADInstruments, Abingdon, Oxon, United Kingdom). TMEM16A blockers were added to the apical side of the epithelium for 5 min before the addition of UTP, that was used to stimulate a TMEM16A-mediated chloride secretory responses.

HBE cultured at air-liquid interface (ALI) for 14–21 days were mounted in Ussing chambers in symmetrical Ringers solution containing (in mM): 120 NaCl, 25 NaHCO_3_, 3.3 KH_2_PO_4_, 0.8 K_2_HPO_4_, 1.2 CaCl_2_, 1.2 MgCl_2_, and 10 glucose, and voltage clamped as described above. In some studies, HBE were pre-treated with IL-13 (10 ng/mL; Peprotech, United Kingdom) for 48 h to increase TMEM16A expression levels (Caputo et al., 2008). Amiloride (10 μM; apical) was used to block the spontaneous, ENaC-mediated current, whilst UTP (10 μM; apical) and forskolin (10 μM; apical and basolateral) were added to the bath solutions to activate Ca^2+^ and cAMP-dependent anion secretory responses, respectively. Inh172 (30 μM; apical and basolateral) was also used to inhibit CFTR function. The TMEM16A blockers were added to the apical side of the Ussing chambers to evaluate effects on each of these currents.

### Intracellular Ca^2+^ measurements

CF-HBE cultured at air-liquid interface for 14–21 days were pre-treated with IL-13 (10 ng/mL; 48 h). CF-HBE were then loaded with the Ca^2+^-sensitive fluorescent reporter dye, Calcium6 for 120 min at 37°C in Hanks balanced salt solution (HBSS) buffered with 20 mM HEPES (pH 7.4). Calcium6 was used as its K_d_ for calcium (320 nM) covers the reported physiological range of [Ca^2+^]_i_ mobilisation by purinoceptors in these cells (0.1–1 μM, Paradiso et al., 2001; Ribeiro et al., 2005). The direct effects of apically administered TMEM16A blockers on [Ca^2+^]_i_ as well as effects on the subsequent responses to UTP were measured using a PHERAstar plate reader (BMG Lab Tech, United Kingdom). Equivalent measurements were performed in FRT-TMEM16Aabc cells that had been cultured in 96 well, clear bottom plastic plates and loaded with Calcium6 using the same protocol.

### Goblet cell formation

HBE cultured at air-liquid interface for 14–21 days were treated with TMEM16A blockers ± IL-13 (10 ng/mL; basolateral) for 96 h to establish any effects on goblet cell numbers. At 48h, LDH levels in the media were measured according to the manufacturer’s instructions (Roche Cytotoxicity Detection Kit #11644793001), and blockers, IL-13 and media were refreshed. At 96 h, wells were fixed in 4% formaldehyde and were stained with antibodies to MUC5AC (45M1; Thermo Fisher, United Kingdom) and acetylated α-tubulin (6-11B-1; Sigma, United Kingdom) as previously described (Danahay et al., 2015; Danahay et al., 2020b) with secondary antibodies to enable fluorescence detection of both proteins (Alexa Fluor; Thermo Fisher, United Kingdom). The MUC5AC^+^ stained area was visualised using a Zeiss Axiovert epifluorescence microscope with a motorised stage that was used to image the same 9 regions of interest on each insert. ImageJ was used to quantify the MUC5AC^+^ and stained area per insert which was normalised to the vehicle control group. The process was repeated for the acetylated α-tubulin^+^ stained area.

### GPCR profiling

Profiling of compound activity *versus* a select panel of diverse GPCRs was undertaken by ThermoFisher Scientific (United Kingdom) *via* their SelectScreen cell-based GPCR assay panel. Detailed experimental protocols on this assay platform can be found at (https://www.thermofisher.com/content/dam/LifeTech/migration/en/filelibrary/services/discovery-research/pdfs.par.36410.file.dat/sscg-brochure.pdf). Briefly these assays detect agonist or antagonist activity using cell lines with the GPCR of interest over-expressed, driving the expression of a reporter gene encompassing a mammalian-optimized Beta-lactamase. Test compounds are incubated with the specific cell lines at the defined final concentration for 5 or 16 h (depending on the cell line specifics) before quantification of effect.

Statistical tests: For comparisons between multiple test compounds and a single vehicle control group, a one-way ANOVA with *post hoc* Dunnett’s test was used with significance assumed when *p* < 0.05.

## Results

### Whole-cell patch clamp assay

**FIGURE 1:**
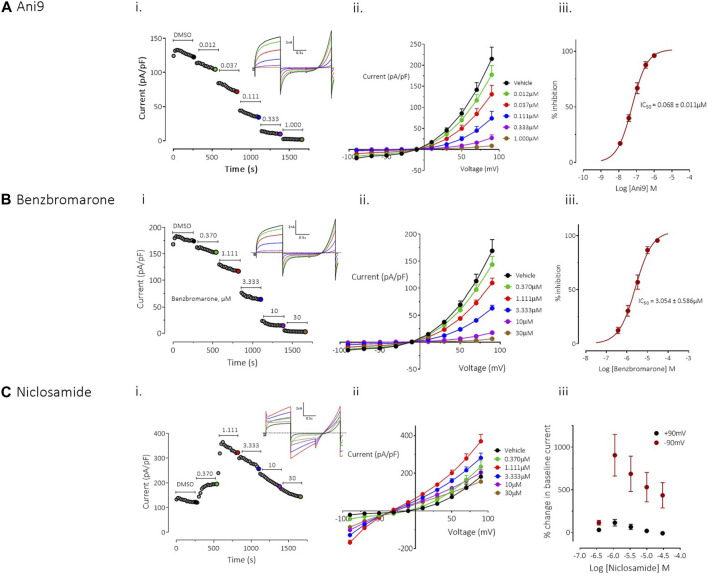
Effects of Ani9, benzbromarone and niclosamide on hTMEM16Aabc currents. Whole-cell patch clamp recordings of the effects of Ani9 **(A)**, benzbromarone **(B)** and niclosamide **(C)** on hTMEM16Aabc currents measured in FRT cells with [Ca^2+^]_i_ clamped at 415 nM. Sample current-time curves (i) for the effects of the indicated compound concentration on peak current at +90mV; inset shows raw current traces for the time points indicated by the coloured dots. Mean current-voltage relationship for each concentration tested (ii) and concentration-response curves (iii) are also shown. Symbols in (ii) and (iii) represent the mean data ±SEM. (*n* = 4–10 experiments in each group). IC_50_ = concentration of test compound required to inhibit 50% of the response.

FRT-TMEM16Aabc cells, with [Ca^2+^]_i_ clamped at 415 nM, displayed large outwardly rectifying currents with slow activation and inactivation kinetics in whole-cell patch clamp recordings (Figure 1), the key biophysical characteristics of TMEM16A. In vehicle (0.3% v/v DMSO) treated cells, the mean peak outward currents at +90 mV slowly declined from 204 ± 30 pA/pF to 150 ± 23 pA/pF (*n* = 7) over the 30 min recording time of the assay (Supplementary Figure S1), which equated to a reduction of 26% ± 3% of the peak current. There was no change in the voltage-dependence of the current over the 30 min recording time, with a similar 23% ± 4% reduction in current observed at −90 mV (−15 ± 1 pA/pF to −11 ± 1 pA/pF, *n* = 7).

Ani9 induced a concentration-dependent inhibition TMEM16Aabc currents with an IC_50_ value of 68 ± 11 nM, as assessed at +90 mV (*n* = 9). The current was almost completely inhibited by Ani9 at 1 µM (93% ± 1% inhibition, *n* = 9; Figure 1A). There was minor voltage dependence to the blockade with the IC_50_ at −90 mV being reduced slightly to 37 ± 3 nM (*n* = 9, *p* < 0.05). Like Ani9, benzbromarone induced a concentration-dependent inhibition of TMEM16Aabc currents (Figure 1B), with an IC_50_ value of 3.0 ± 0.6 µM (*n* = 10), as assessed at +90 mV. As with Ani9, benzbromarone inhibition was slightly more potent when the assessed at −90 mV (IC_50_ = 1.3 ± 0.3 µM, *n* = 10, *p* < 0.05). Benzbromarone inhibited the TMEM16Aabc current by 96% ± 1% (*n* = 10) at the highest concentration tested (30 µM).

In contrast to the clear inhibitory effects of Ani9 and benzbromarone on TMEM16Aabc currents, niclosamide produced a “bell-shaped” response, with potentiation of currents at concentrations up to 1.1 µM (Figure 1C; *n* = 4). At higher concentrations of niclosamide, the increase in current magnitude was reduced. The effects of niclosamide were more pronounced on the inward than the outward currents such that the rectification ratio (ratio of peak outward current at +90 mV to the peak inward current at −90 mV) was reduced from 10.0 ± 1.0 in the presence of vehicle alone, to 2.3 ± 0.3 (*n* = 4, *p* < 0.05) by 1.1 µM niclosamide. The change in rectification was also accompanied by a leftward shift in the current reversal potential from −12.3 ± 2.0 mV with vehicle, to −29.8 ± 1.3 mV (*n* = 4, *p* < 0.05) in the presence of 30 µM niclosamide.

To provide additional insight into the unexpected profile, the effects of niclosamide were evaluated on an additional TMEM16A isoform, TMEM16Aacd. The effects of niclosamide on TMEM16Aacd, stably expressed in HEK cells, with [Ca^2+^]_i_ clamped at 415 nM, were identical to those observed in the FRT-TMEM16Aabc cells, i.e., a “bell shaped” concentration response, with enhancement of currents at low µM accompanied by loss of rectification and a left shift in the current reversal potential (see Supplementary Figure S2A). When [Ca^2+^]_i_ in the cells was clamped to 0 nM to remove activation of the TMEM16Aacd channels, the cells exhibited small (<5 pA/pF) currents that lacked the biophysical characteristics of TMEM16A, i.e., no outward rectification and time-dependent activation/inactivation (Supplementary Figure S2B). However, under these conditions niclosamide induced a clear concentration-dependent increase in current with an EC_50_ value of 2.7 ± 0.3 µM (*n* = 6). The current retained linear rectification characteristics, lacked time dependent activation or inactivation kinetics and reversed at a more hyperpolarised reversal potential than the baseline current of approximately −50 mV. Quantitatively similar effects were induced by niclosamide on the parental HEK cell line, which lacked expression of TMEM16A, suggesting that niclosamide can induce activation of a non-TMEM16A conductance in these cells (Supplementary Figure S3).

### Ion transport and [Ca^2+^]_i_ measurements

**FIGURE 2:**
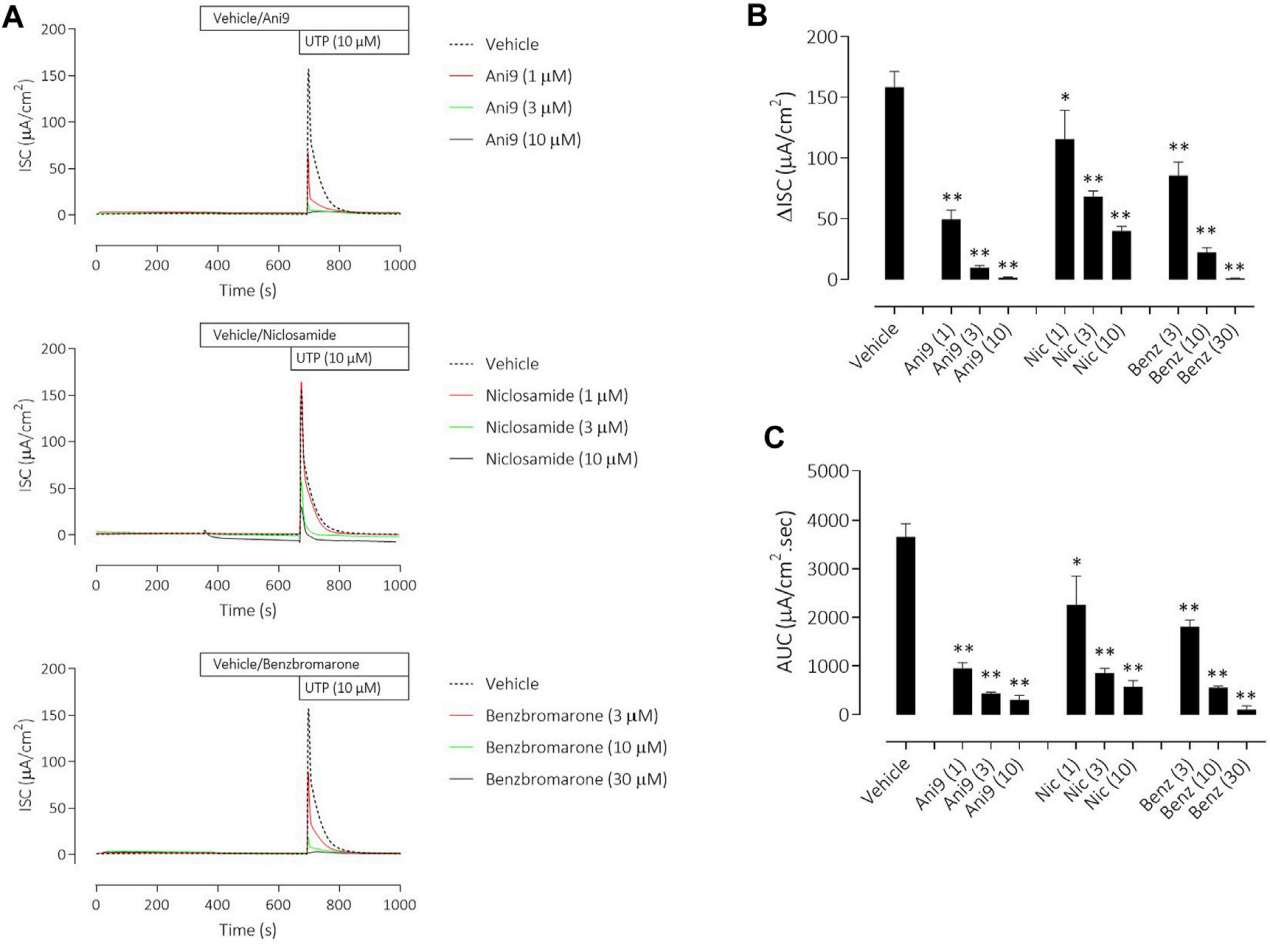
Ani9, niclosamide and benzbromarone attenuate UTP-stimulated anion secretion in FRT-TMEM16Aabc monolayers. Sample short-circuit current traces illustrating the effects of Ani9, niclosamide (Nic) and benzbromarone (Benz) on the apical UTP-stimulated response of FRT-TMEM16Aabc monolayers **(A)**. The peak increase in ISC **(B)** as well as the integrated AUC **(C)** were measured. Mean data ±SEM (*n* = 8–11 inserts per group) are shown. Concentrations of test compounds are shown in brackets as µM. * and ** denote *p* < 0.0002 and *p* < 0.0001 respectively following a one-way ANOVA using *post hoc* Dunnett’s test.

**FIGURE 3:**
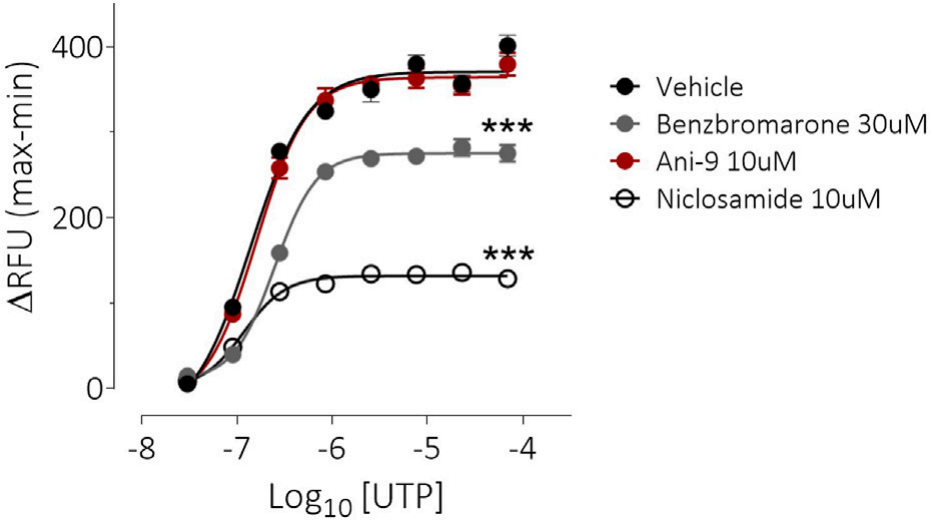
Benzbromarone and niclosamide affect UTP-stimulated calcium mobilisation in FRT-hTMEM16Aabc cells. Mean data ±SEM illustrating the effects of a 10 min incubation of FRT-hTMEM16Aabc cells with test compounds on a subsequent UTP-stimulated increase in [Ca^2+^]_i_. Cells had been loaded with Calcium6 dye and were stimulated with UTP (0.03–69 µM) and the changes in fluorescence recorded. Peak changes in fluorescence minus the baseline levels are plotted for each compound. *** denotes *p* < 0.001 (*n* = 6 experiments per groups).

 Under an imposed basolateral to apical chloride gradient, the addition of UTP (10 µM) to the apical side of FRT-TMEM16Aabc monolayers induced a transient increase in ISC (Figure 2) of 158.4 ± 13.0 µAcm^-2^ (*n* = 11). Treatment with either Ani9, benzbromarone or niclosamide for 5 min attenuated the subsequent UTP-stimulated peak increase in ISC in addition to the integrated ISC response (AUC) in a concentration-dependent manner (*p* < 0.0001; *n* = 8–11). Of note, Ani9 and benzbromarone were without effect on the baseline ISC prior to the addition of UTP. In contrast, niclosamide (10 µM) induced a bi-phasic response immediately on addition to the cells. Initially niclosamide stimulated a transient increase in ISC (4.4 ± 0.3 µAcm^-2^ compared to 0.0 ± 0.1 µAcm^-2^ in the vehicle control; *p* < 10^–5^) and a subsequent attenuation of the baseline current (−6.7 ± 0.7 µAcm^-2^ compared to −0.3 ± 0.1 µAcm^-2^ in the vehicle control; *p* < 10^–4^). In separate experiments, the UTP-stimulated increase in [Ca^2+^]_i_ in FRT-TMEM16Aabc cells was unaffected by pre-treatment with Ani9 (Figure 3). In contrast, both benzbromarone and niclosamide significantly attenuated the maximal UTP-stimulated elevation of [Ca^2+^]_i_.

**FIGURE 4:**
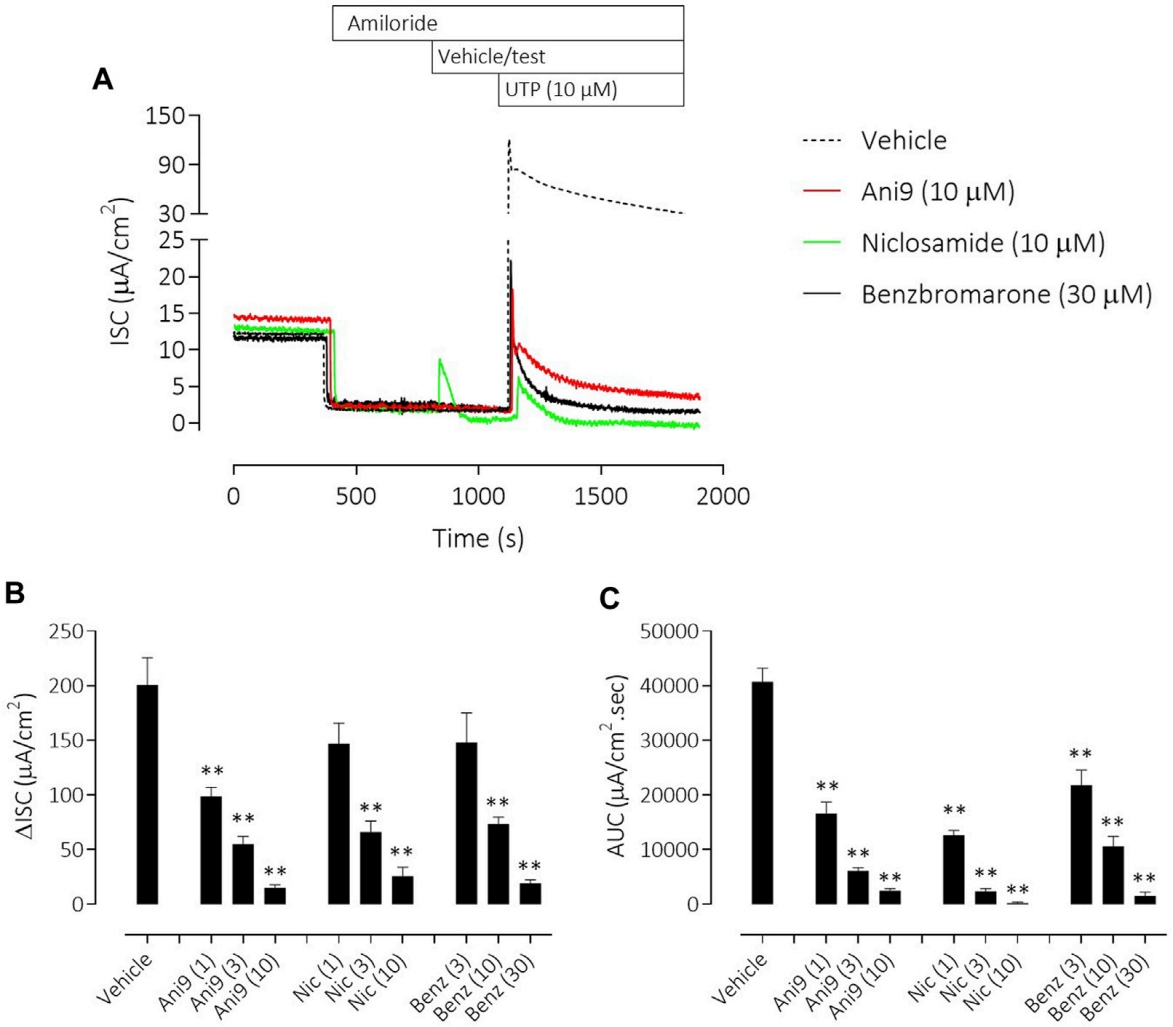
Ani9, niclosamide and benzbromarone attenuate UTP-stimulated anion secretion in CF-HBE cells. Sample short-circuit current traces illustrating the effects of Ani9, niclosamide (Nic) and benzbromarone (Benz) on the apical UTP-stimulated response of IL-13 pre-treated CF-HBE cells **(A)**. The peak increase in ISC **(B)** as well as the integrated AUC **(C)** were measured. Mean data ±SEM (*n* = 3-5 inserts per group) are shown. Concentrations of test compounds are shown in brackets as µM. ** denotes *p* < 0.0001 respectively following a one-way ANOVA using *post hoc* Dunnett’s test.

**FIGURE 5:**
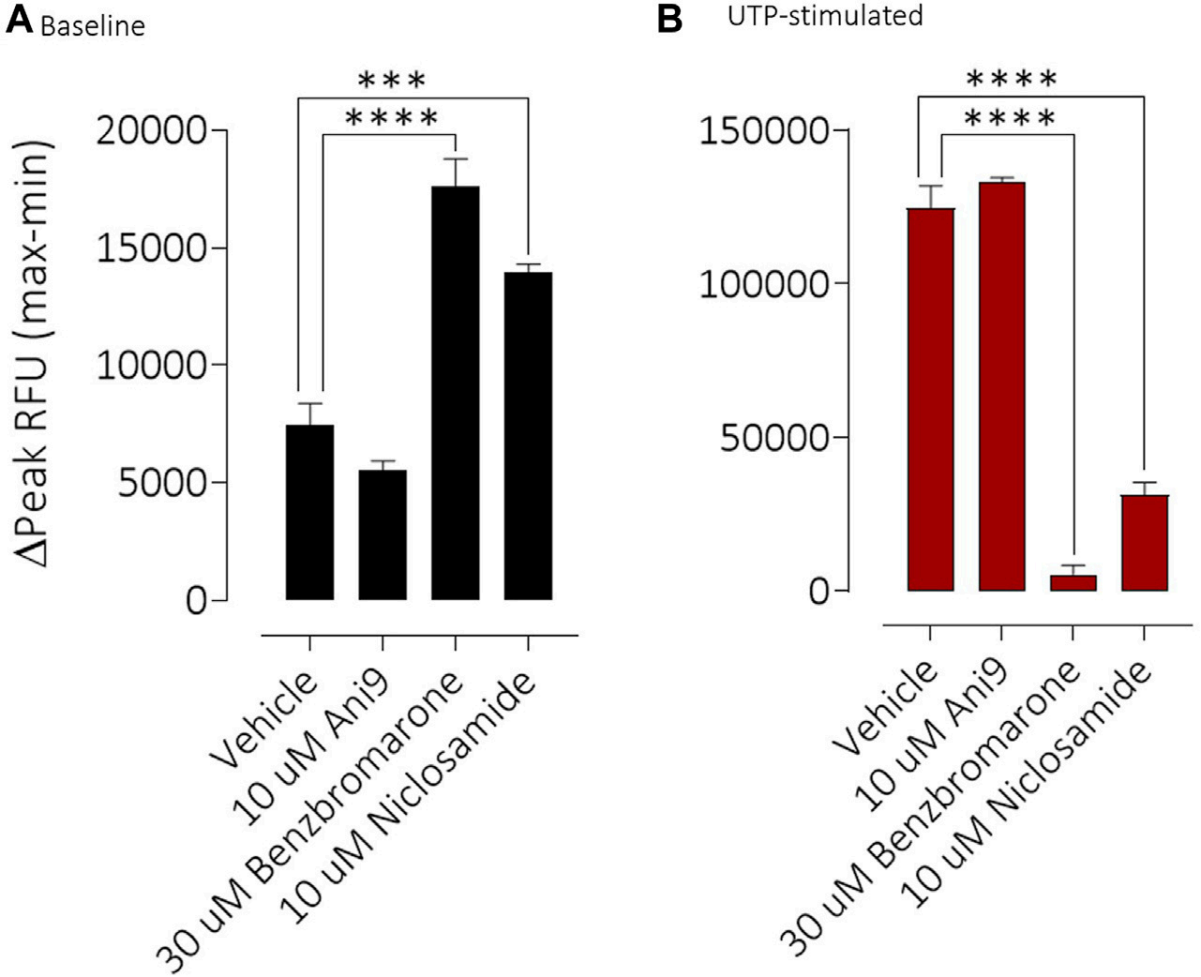
Benzbromarone and niclosamide affect calcium handling in differentiated cystic fibrosis human bronchial epithelial (HBE) cells. Mean data ±SEM illustrating the direct effects of Ani9, benzbromarone and niclosamide on [Ca^2+^]_i_ **(A)** and also on the increases in [Ca^2+^]_i_ stimulated by a maximal concentration of UTP **(B)** in CF-HBE. Peak changes in fluorescence minus the baseline levels are plotted for the effects of each compound under both conditions. Mean data ±s. e.m. (4–10 inserts per group). *** and **** denote *p* < 0.001 and *p* < 0.0001 respectively.

Similar results were obtained with the three TMEM16A blockers in IL-13 pre-treated CF-HBE (Figure 4). After the ENaC-mediated ISC had been blocked with amiloride, Ani9 and benzbromarone treatment for 5 min attenuated the subsequent UTP-stimulated increase in ISC, quantified as both the peak increase in ISC and AUC that was concentration-dependent (*p* < 0.0001; *n* = 3–5). Neither Ani9 nor benzbromarone had any direct effect on the amiloride-insensitive baseline ISC when added to the cells. In contrast, niclosamide (10 µM) induced an immediate, transient increase in ISC (6.8 ± 1.0 µAcm^-2^ compared to 0.5 ± 0.2 µAcm^-2^ in the vehicle control; *p* < 0.001) and returned to a lower baseline ISC than before compound addition (−0.9 ± 0.2 µAcm^-2^ compared to −0.3 ± 0.1 µAcm^-2^ in the vehicle control; *p* = 0.02). The subsequent UTP-stimulated increase in ISC was also attenuated by niclosamide. In separate experiments, but again using IL-13 pre-treated CF-HBE cultured at ALI, neither baseline [Ca^2+^]_i_ or the UTP-stimulated increase in [Ca^2+^]_i_ was affected by pre-treatment with Ani9 (Figure 5). In contrast, both benzbromarone and niclosamide induced an immediate and transient increase in baseline [Ca^2+^]_i_ when added to the epithelium and attenuated the subsequent UTP-stimulated elevation of [Ca^2+^]_i_.

**FIGURE 6:**
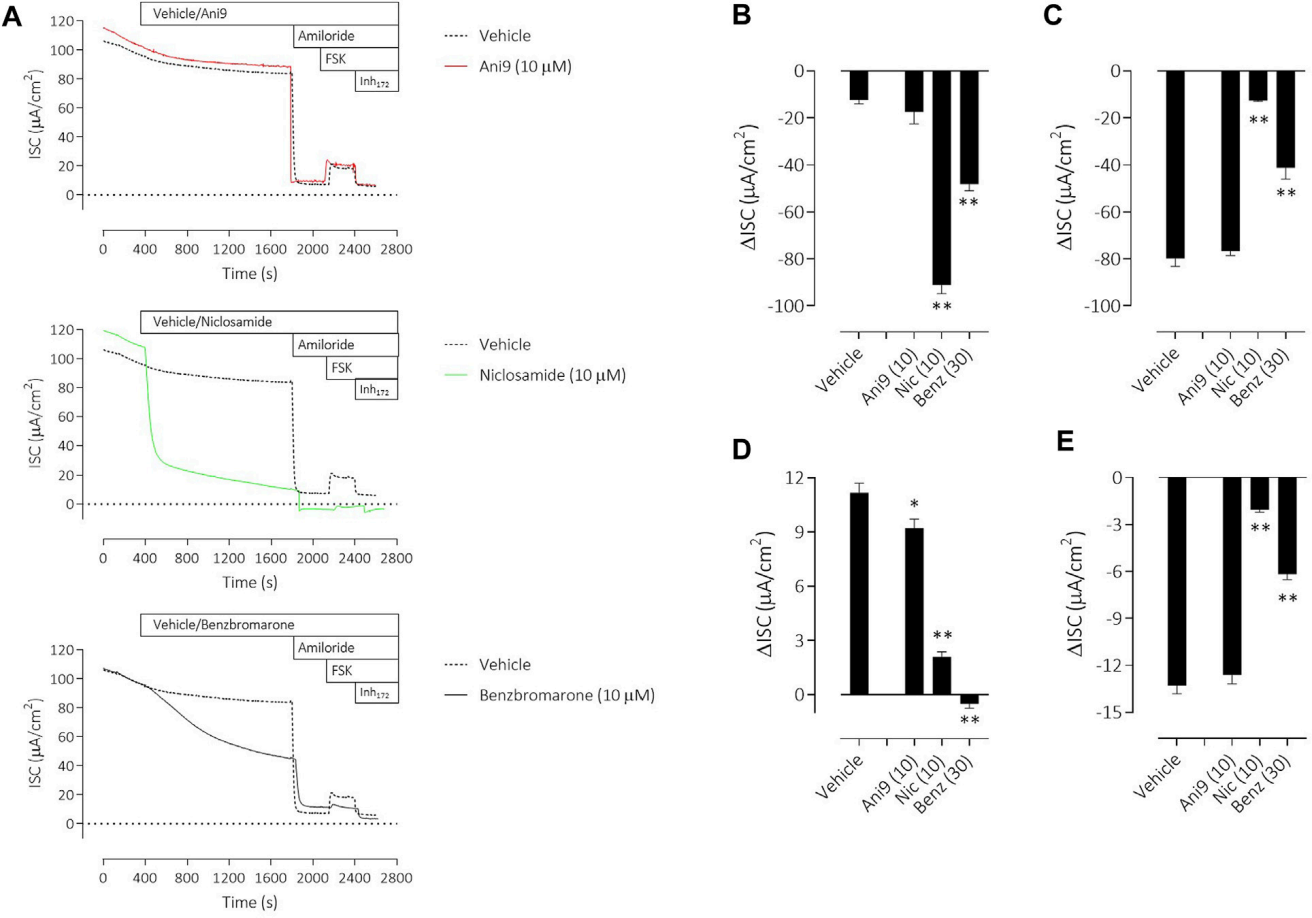
Multiple effects of niclosamide and benzbromarone on epithelial ion transport mechanisms in HBE cells. Sample short-circuit current traces **(A)** illustrating the effects of Ani9, niclosamide (Nic) and benzbromarone (Benz) on the ion transport properties of HBE cells (non-CF). The mean ± SEM changes in baseline **(B)**, amiloride-sensitive **(C)**, forskolin-stimulated **(D)** and Inh172-sensitive **(E)** ISC responses are shown (*n* = 6 inserts per group). Concentrations of test compounds are shown in brackets as µM. * and ** denote *p* < 0.01 and *p* < 0.0001 respectively following a one-way ANOVA using *post hoc* Dunnett’s test.

To understand the specificity of these TMEM16A blockers in HBE, Ani9, benzbromarone and niclosamide were added to the apical side of the epithelium at the start of the protocol and before any other manoeuvres. Non-CF HBE were used for these studies to enable an evaluation of both the ENaC and CFTR mediated ion transport processes. Ani9 (10 µM) had no effect on: 1) the baseline ISC or transepithelial resistance, 2) on the magnitude of the subsequent response to amiloride or 3) on the forskolin stimulated, Inh172-sensitive ISC, when compared with the vehicle (Figure 6A). There was a small but significant inhibitory effect of Ani9 on the peak increase in forskolin-stimulated current. In contrast, niclosamide (10 µM) induced an approximate 90% reduction in the baseline ISC within 30 min of addition to the cells (Figure 6B), that was associated with a significant attenuation of the transepithelial resistance (−174 ± 19 Ω cm^2^ *versus* +108 ± 14 Ω cm^2^ in the vehicle control; *p* < 10^–6^, *n* = 6). The subsequent amiloride-sensitive, forskolin-stimulated and Inh172-sensitive currents were all also attenuated after niclosamide treatment. Benzbromarone induced a similar, 50% decline in the baseline ISC (Figure 6C) but tended to increase the transepithelial resistance (+170 ± 25 Ω cm^2^ *versus* +108 ± 14 Ω cm^2^ in the vehicle control; *p* = 0.053, *n* = 6). Benzbromarone treatment also attenuated the subsequent amiloride-sensitive, forskolin-stimulated and Inh172-sensitive currents.

### Effect of TMEM16A inhibitors on goblet cell formation

**FIGURE 7:**
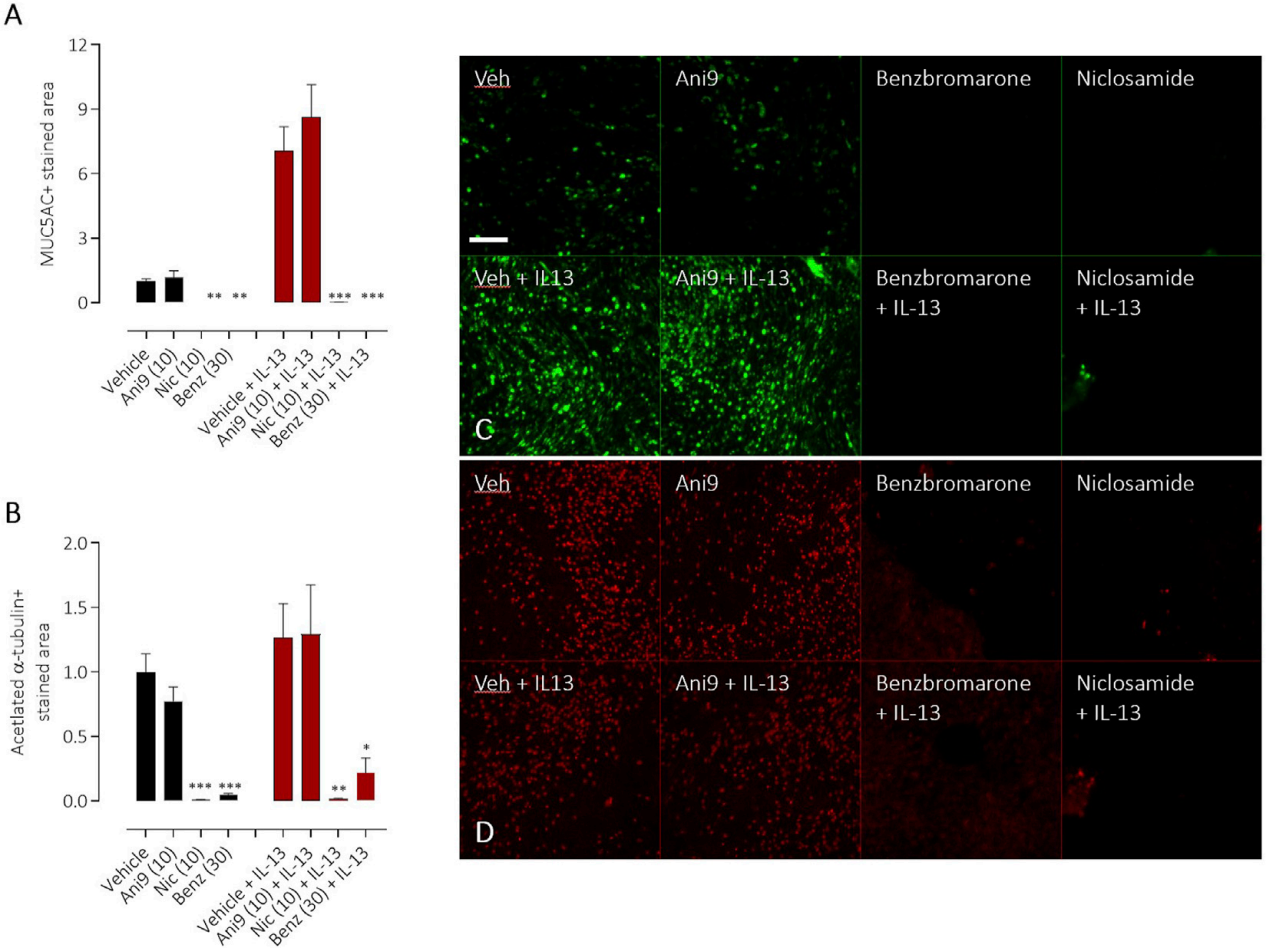
Effects of TMEM16A blockers on IL-13 stimulated goblet cell formation in HBE. The effects of Ani9, niclosamide (Nic) and benzbromarone (Benz) on normal (black bars) or IL-13 treated (red bars) HBE cultures. Mean ± SEM stained areas of MUC5AC + goblet cells **(A)** and acetylated α-tubulin + multiciliated cells **(B)** are shown (*n* = 5 inserts per group). Concentrations of test compounds are shown in brackets as µM. *, ** and *** denote *p* < 0.02, *p* < 0.006 and *p* < 0.0002 respectively following a one-way ANOVA using *post hoc* Dunnett’s test. Sample immunofluorescence images are shown illustrating the respective staining for MUC5AC+ goblet cells **(C)** and acetylated α-tubulin+ multiciliated cells **(D)**.

TMEM16A inhibitors have been widely reported to influence the differentiation of airway epithelial cells and to specifically attenuate the formation of mucin producing goblet cells. Using HBE in an assay format to quantify goblet cell formation, IL-13 treatment for 96 h induced a significant, 7.0 ± 1.1 fold increase in the number of goblet cells (Figure 7A), measured as the increase in MUC5AC+ stained area (*p* < 0.001, *n* = 5). Concurrent treatment with Ani9 (10 μM; 96 h) did not affect the MUC5AC+ stained area in either the IL-13 naïve or IL-13 treated cells. There was likewise no effect of Ani9 on the acetylated α-tubulin (ciliated) stained area (Figure 7B). In contrast, treatment of HBE with either niclosamide or benzbromarone significantly attenuated the MUC5AC+ stained area of naïve and IL-13 exposed cultures. These treatments also significantly attenuated the acetylated α-tubulin-stained area. It was observed in these experiments that by 48 h after initiating treatment of HBE, the air-liquid interface was compromised in the niclosamide treated group, with media observed on to the mucosal surface of the cells. Analysis of basolateral media collected at this 48 h timepoint revealed detectable levels of LDH (0.098 ± 0.003 U/mL) in the niclosamide treated group compared to undetectable levels in the other treatment groups.

### GPCR selectivity profiling

When profiled against a small panel of 22 functional GPCR assays (Supplementary Figure S4), Ani9 (10 µM) inhibited 2 GPCR targets by ≥ 50% (5-HT2B, delta opioid). In contrast benzbromarone and niclosamide (10 µM), inhibited 6 and 11 GPCRs respectively by ≥ 50%.

## Discussion

Inhibiting TMEM16A function has been proposed as a novel approach to treat several respiratory diseases including asthma, cystic fibrosis, COVID-19 infection and pulmonary arterial hypertension (PAH) (Cabrita et al., 2019; Kunzelmann et al., 2019; Miner et al., 2019; Papp et al., 2019; Singh et al., 2022). Benzbromarone and niclosamide have been used clinically as a uricosuric agent and anti-helmintic respectively and have been more recently described as blockers of TMEM16A (Huang et al., 2012; Miner et al., 2019). To this end, both niclosamide and benzbromarone have entered clinical trials as repurposed therapies for the treatment of some of these respiratory conditions (ClinicalTrials.gov NCT04644705; Papp et al., 2019) and in view of this it is surprising that data supporting their selectivity and even mechanism of action on TMEM16A are limited. The most illuminating data in the present study, is the observation that niclosamide does not block TMEM16A but rather attenuates channel activity through an indirect inhibitory effect on intra-cellular Ca^2+^ signalling. Benzbromarone does block TMEM16A activity, but together with niclosamide, shares a number of TMEM16A-independent activities that complicate the interpretation of preclinical and now clinical studies.

In patch-clamp experiments using conditions where [Ca^2+^]_i_ was tightly clamped and therefore changes in current were independent of changes in [Ca^2+^]_i_ (Bill et al., 2015; Danahay et al., 2020a), benzbromarone and Ani9 both inhibited TMEM16Aabc activity. Effects of these inhibitors were similar on both inward and outward currents and displayed profiles consistent with a *bona fide* block of channel activity. In contrast, niclosamide failed to inhibit the activity of both TMEM16Aabc and TMEM16Aacd isoforms, paradoxically potentiating the current at concentrations ≤1 μM and induced a significant leftward shift in the reversal potential. In view of the niclosamide-induced activation of a current with [Ca^2+^]_i_ clamped to 0 nM in both HEK-TMEM16Aacd cells as well as in the parental HEK cell, it is likely that niclosamide activates an additional, non-TMEM16A conductance(s) in both the HEK and FRT cell lines.

Niclosamide has however previously been reported to block TMEM16A in patch-clamp experiments (Centeio et al., 2020). In the present studies niclosamide attenuated UTP-stimulated increases in short circuit current in monolayers of both FRT-TMEM16Aabc and CF-HBE. In each of these assay formats where niclosamide has demonstrated an inhibition of TMEM16A activity [Ca^2+^]_i_ has been unbuffered and is thus under normal physiological control. We hypothesised that niclosamide may be affecting the physiological regulation [Ca^2+^]_i_ signalling leading to the artefactual observation and description of niclosamide as a TMEM16A blocker. Consistent with this hypothesis, niclosamide attenuated the UTP-stimulated increase in [Ca^2+^]_i_ in both FRT-TMEM16Aabc and CF-HBE. In contrast, Ani9 did not affect the UTP-stimulated rise in [Ca^2+^]_i_. These data support the concept that niclosamide does not block TMEM16A directly, but does attenuate [Ca^2+^]_i_ in response to purinergic stimulation. Ani9 blocks TMEM16A but importantly, has no influence on [Ca^2+^]_i_. Benzbromarone shared characteristics of both niclosamide and Ani9 in that it both blocked TMEM16A activity under conditions of [Ca^2+^]_i_ buffering but also attenuated stimulated [Ca^2+^]_i_ responses. Similar data confirming the effect of niclosamide on [Ca^2+^]_i_ and the lack of effect of Ani9 have been recently reported (Genovese et al., 2022) and suggest that niclosamide may be inhibiting the SERCA pump. An inhibitory effect of niclosamide on the SERCA pump would also be consistent with the transient increase in ISC that was observed in both FRT-TMEM16A and HBE ion transport experiments (Figures 2, 4).

To further evaluate the selectivity profiles of niclosamide, benzbromarone and Ani9, compounds were added to non-CF HBE under voltage clamp conditions to examine potential effects on ENaC and CFTR-dependent ion transport processes. Ani9 was without effect on either the baseline or amiloride-sensitive currents. The small but significant effect of Ani9 on the forskolin stimulated ISC likely reflects a component of this current being TMEM16A-mediated rather than an effect on CFTR directly as this compound has previously been shown to not affect CFTR function (Seo et al., 2016) and is further supported by the lack of effect on the Inh172-sensitive current which is CFTR-mediated (Figure 6E). In contrast, both niclosamide and benzbromarone significantly attenuated the baseline, ENaC-mediated and CFTR currents. In addition, niclosamide significantly reduced the transepithelial resistance whilst benzbromarone induced a trend towards a tightening of the epithelium (*p* = 0.053). It would appear unlikely that both niclosamide and benzbromarone are direct blockers of ENaC and CFTR. It is more likely that both compounds have a generalised effect on active ion transport processes secondary to the previously described effects on [Ca^2+^]_i_ homeostasis or perhaps due to activity of these agents on mitochondrial function (Felser et al., 2014; Tao et al., 2014). The relative selectivity of the three compounds was also assessed using a small panel of 22 GPCR assays. Ani9 inhibited 2 GPCR targets when tested at a concentration >130-fold over it is IC_50_ for TMEM16A. In contrast, benzbromarone inhibited six GPCR targets at 4x it is IC50 for TMEM16A whilst niclosamide inhibited 11 GPCRs, that would be indicative of a greater potential for off-target effects in biological systems compared with Ani9.

Finally, we evaluated the effects of these compounds on cellular differentiation in HBE following IL-13 treatment. IL-13 both increases the expression of functional TMEM16A and promotes the formation of MUC5AC+ goblet cells (Caputo et al., 2008; Danahay et al., 2015) and previous studies have proposed a mechanism whereby increased TMEM16A channel activity drives goblet cell formation (Lin et al., 2015; Qin et al., 2016; Kondo et al., 2017; Benedetto et al., 2019). This proposed activity of TMEM16A has been used as one of the salient arguments to progress TMEM16A blockers as novel therapeutics to treat mucus hypersecretion in asthma and CF (Benedetto et al., 2019; Cabrita et al., 2019). As previously reported, Ani9 was without effect on the numbers of MUC5AC+ goblet cells or acetylated α-tubulin+ ciliated cells under both naïve and IL-13 stimulated conditions (Danahay et al., 2020b). In contrast, niclosamide and benzbromarone treatment attenuated numbers of both goblet and ciliated cells irrespective of IL-13 treatment. These data do not however support a role for TMEM16A channel function as a regulator of cellular differentiation but rather suggest that previous reports to the contrary that used either benzbromarone or niclosamide (Kondo et al., 2017; Benedetto et al., 2019) are likely due to a non-selective, potentially toxic effect of these agents. The broad effect of both niclosamide and benzbromarone on active ion transport processes in these cells, the increase in LDH release (niclosamide only) and loss of epithelial integrity, together with published effects on mitochondrial function further support this proposal.

In view of these data, the published studies supporting the repurposing of niclosamide and benzbromarone as TMEM16A blocker therapies for respiratory diseases should be carefully reviewed. In addition to the data reported in the present study, niclosamide has been demonstrated to relax freshly isolated human bronchial smooth muscle and on this basis proposed TMEM16A blockade as a novel bronchodilator mechanism (Miner et al., 2019). This effect of niclosamide has been confirmed but also demonstrated to be an effect that is not shared by Ani9 (Danahay et al., 2020b). Furthermore, ETX001, a recently described TMEM16A potentiator shows no effect on freshly isolated human bronchial smooth muscle tone or on lung function *in vivo*. Together, these data suggest that niclosamide is likely relaxing airway smooth muscle through an effect on [Ca^2+^]_i_ that is independent of TMEM16A. Benzbromarone has similarly been demonstrated to relax vascular smooth muscle *in vitro* through a proposed TMEM16A blocking mechanism (Papp et al., 2019). It is however noteworthy that the structurally diverse TMEM16A inhibitors T16Ainh-A01 and Ani9 (Papp et al., 2019; Danahay et al., 2020b) failed to show activity in human vascular smooth muscle preparations, that would support an off-target effect of benzbromarone as having driven the vasodilator responses. Of note, when subsequently evaluated in a small clinical study in PAH patients, benzbromarone induced a paradoxical increase in pulmonary artery pressure (Papp et al., 2019).

In summary, these data do not support the continued use or description of niclosamide as a blocker of TMEM16A and historical reports to this end should be interpreted with caution. Benzbromarone can be considered as a TMEM16A blocker, although the potential for off-target effects on [Ca^2+^]_i_ should be carefully considered during experimental design and in the interpretation of historical data. Based on available data, Ani9 is presently the most potent and selective TMEM16A inhibitor, displaying selectivity over related family members.

## Data Availability

The raw data supporting the conclusion of this article will be made available by the authors, without undue reservation.
