# Supplementary material for: Niclosamide does not modulate airway epithelial function through blocking of the calcium activated chloride channel, TMEM16A
Source: Front Pharmacol. 2023 Mar 6;14:1142342. doi: 10.3389/fphar.2023.1142342 (PMC10025480; doi:10.3389/fphar.2023.1142342)
Supplement: Supplementary file 1 [file DataSheet1.docx]

**Niclosamide does not modulate airway epithelial function through blocking of the calcium activated chloride channel, TMEM16A**

**Supplemental information**

Henry Danahay^1*^, Sarah Lilley^2^, Kathryn Adley^2^, Holly Charlton^2^, Roy Fox^2^ and Martin Gosling^1^

^1^Enterprise Therapeutics Ltd. 60 Science Park Square, Brighton BN1 9SB, UK

^2^Sussex Drug Discovery Centre, School of Life Sciences, University of Sussex, Brighton, BN1 9RH, UK

^*^Corresponding author

Work was funded by Enterprise Therapeutics Ltd, UK

**Supplemental results**


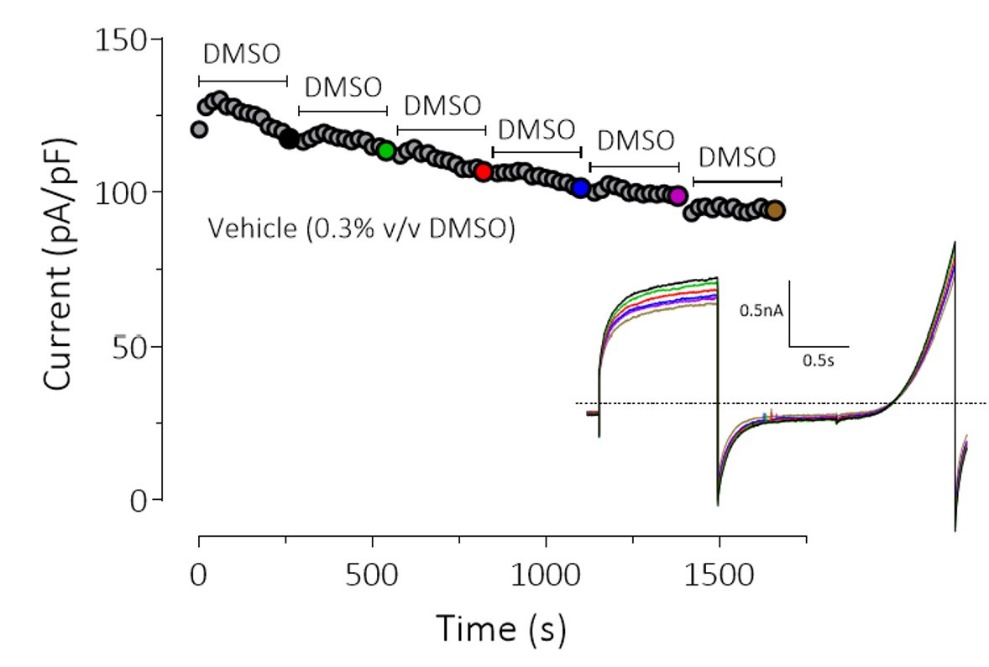


**Figure S1 Effects of vehicle (0.1% DMSO) on hTMEM16Aabc currents**

Whole-cell patch clamp recordings of the effects of 0.1% v/v DMSO on hTMEM16Aabc currents measured in FRT cells with [Ca^2+^]_i_ clamped at 415nM. Main figure shows current-time curve for the effects of 6 additions of 0.1% v/v DMSO on peak current at +90mV; inset shows raw current traces for the time points indicated by the coloured dots. Data from a single cell representative of 6 others.


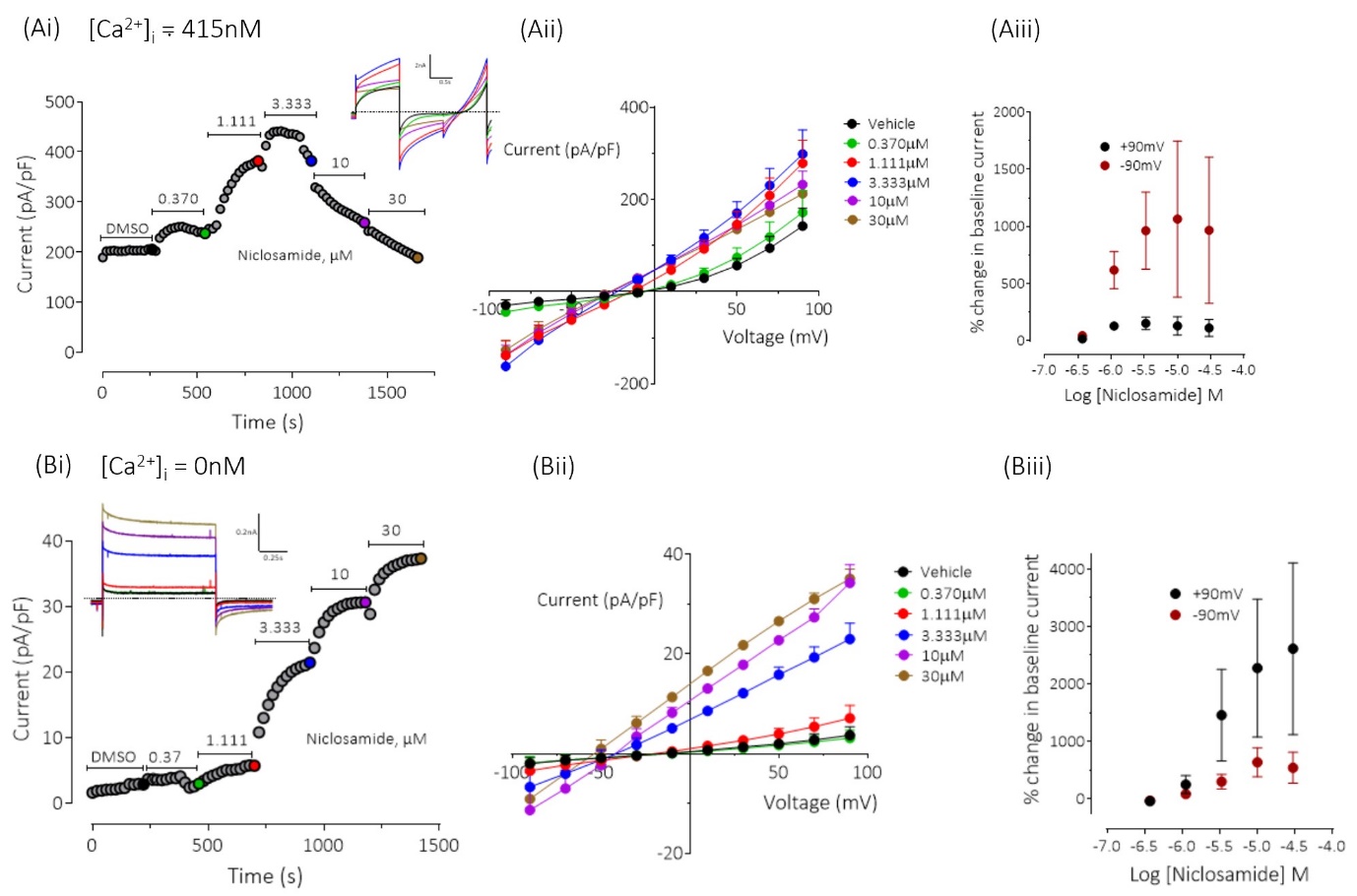


**Figure S2 Effects of niclosamide on hTMEM16Aacd currents**

Whole-cell patch clamp recordings of the effects of niclosamide on hTMEM16Aacd currents measured in HEK cells with [Ca^2+^]_i_ clamped at (A) 415 nM (n=5) or (B) 0 nM (n=6). Sample current-time curves (i) for the effects of the indicated compound concentration on peak current at +90mV; inset shows raw current traces for the time points indicated by the coloured dots. Mean ± SEM current-voltage relationship data for each concentration tested (ii) and associated concentration-response curves (iii) are shown (n=5 and 4 experiments under conditions (A) and (B) respectively).


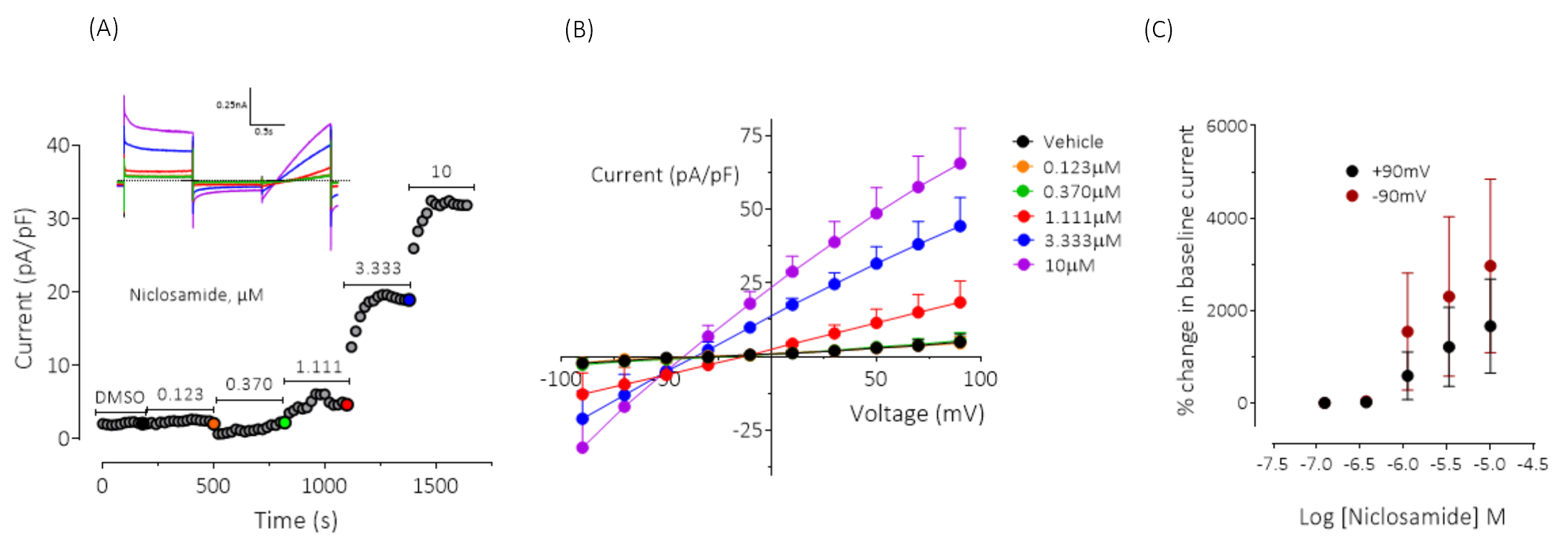


**Figure S3 Effects of niclosamide on currents in HEK null cells**

Whole-cell patch clamp recordings of the effects of niclosamide on currents measured in HEK nulls cells with [Ca^2+^]_i_ clamped at 415nM. Sample current-time curve for the effects of the indicated concentration on peak current at +90mV (A); inset shows raw current traces for the time points indicated by the coloured dots. Mean ± SEM (n=4) current-voltage relationship for each concentration tested (B) and associated concentration-response curves (C) are shown.


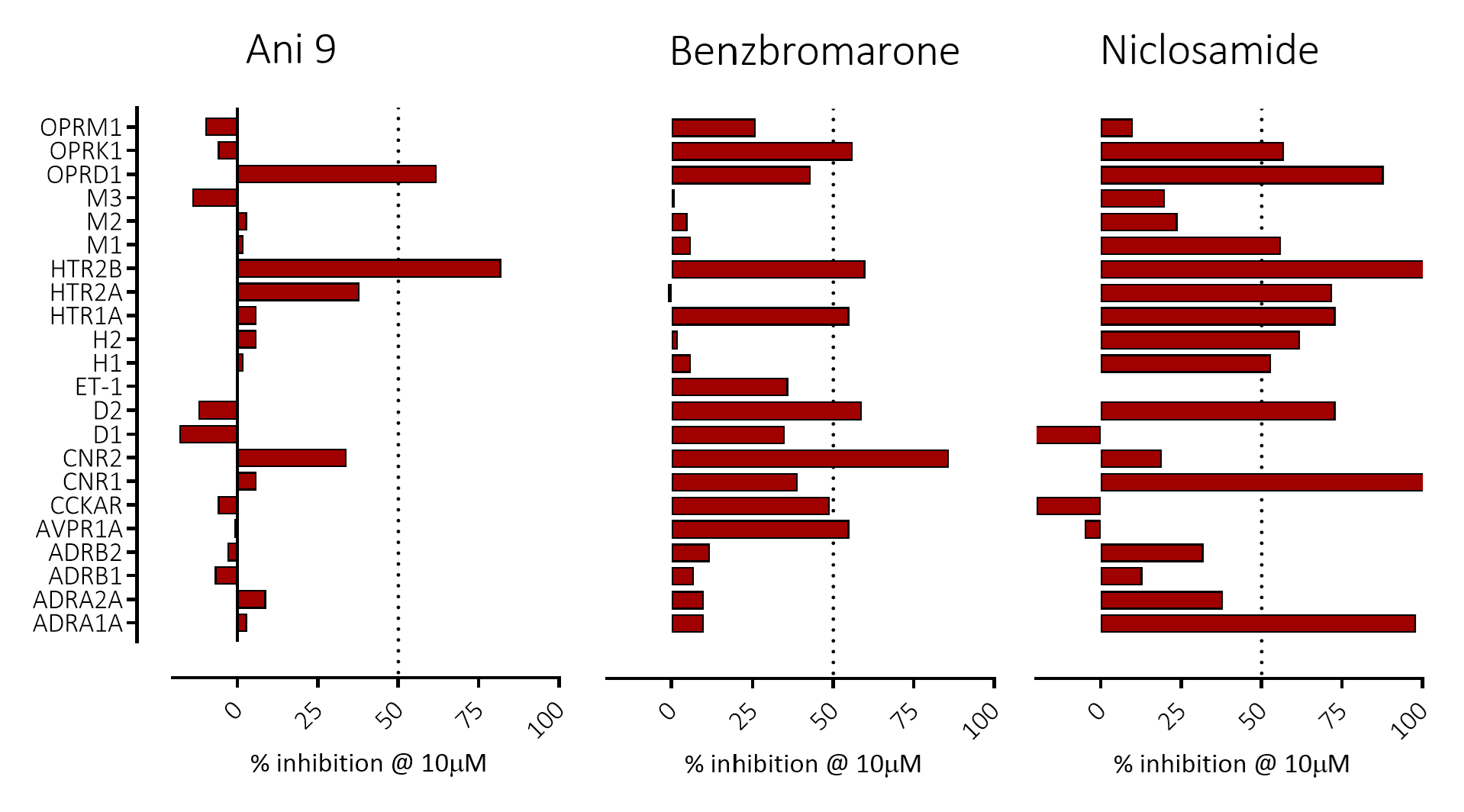


**Figure S4 Selectivity profile of Ani9, benzbromarone and niclosamide versus a select panel of G-protein coupled receptors**

Compounds were tested against a panel of GPCRs to assess their selectivity profiles at a single concentration of 10 µM. Bars show the mean effects for each receptor from a duplicate determination.
